# Dietary analysis of an uncharacteristic population of the Mountain Pygmy-possum *(Burramys parvus)* in the Kosciuszko National Park, New South Wales, Australia

**DOI:** 10.7717/peerj.6307

**Published:** 2019-01-24

**Authors:** Tahneal Hawke, Hayley Bates, Suzanne Hand, Michael Archer, Linda Broome

**Affiliations:** 1 PANGEA Research Centre, Biological, Earth and Environmental Sciences, University of New South Wales, Kensington, NSW, Australia; 2 Office of Environment and Heritage, Queanbeyan, NSW, Australia

**Keywords:** Diet, Mountain Pygmy-possum, Kosciuszko, *Burramy parvus*, Dietary analysis

## Abstract

**Background:**

The Mountain Pygmy-possum (*Burramys parvus*) is a critically endangered marsupial, endemic to alpine regions of southern Australia. We investigated the diet of a recently discovered population of the possum in northern Kosciuszko National Park, NSW, Australia. This new population occurs at elevations well below the once-presumed lower elevation limit of 1,600 m.

**Goals and Methods:**

Faecal material was analysed to determine if dietary composition differed between individuals in the newly discovered northern population and those in the higher elevation southern population, and to examine how diet was influenced by rainfall in the southern population and seasonal changes in resource availability in the northern population.

**Results and Discussion:**

The diet of *B. parvus* in the northern population comprised of arthropods, fruits and seeds. Results indicate the diet of both populations shares most of the same invertebrate orders and plant species. However, in the absence of preferred food types available to the southern population, individuals of the northern population opportunistically consumed different species that were similar to those preferred by individuals in higher altitude populations. Differing rainfall amounts had a significant effect on diet, with years of below average rainfall having a greater percentage composition and diversity of invertebrates. Seasonal variation was also recorded, with the northern population increasing the diversity of invertebrates in their diet during the Autumn months when Bogong Moths (*Agrotis infusa*) were absent from those sites, raising questions about the possum’s dependence on the species

**Conclusions:**

Measurable effects of rainfall amount and seasonal variation on the dietary composition suggest that predicted climatic variability will have a significant impact on its diet, potentially impacting its future survival. Findings suggest that it is likely that *B. parvus* is not restricted by dietary requirements to its current pattern of distribution. This new understanding needs to be considered when formulating future conservation strategies for this critically endangered species.

## Introduction

Globally, Australia has the highest rate of recent native mammal extinctions, with 22 extinct species and 26 species remaining as only remnant populations (Short & Smith, 1994; Woinarski et al., 2011). Fourteen percent of Australia’s native rodents and six percent of marsupials have become extinct since European settlement (Woinarski et al., 2011). In particular, small mammals have been under increasing threat, with  Cardillo & Bromham (2001) reporting that small mammals in the critical weight range of 35–5,500 g are particularly susceptible to extinction. Additionally, climate change has been identified as a major threat to many of Australia’s native species, with many species expected to undergo range reductions as a result of a warming climate (Brereton, Bennett & Mansergh, 1995).

The Mountain Pygmy-possum (*Burramys parvus*) is a small terrestrial Australian marsupial endemic to high elevation boulder fields in the Australian Alps bioregion of eastern Australia. It is currently listed as critically endangered by the IUCN red list of threatened species (Körtner & Geiser, 1995; Schulz, Wilks & Broome, 2012a; Schulz, Wilks & Broome, 2012b; Menkhorst et al., 2013). Populations have been in decline since the discovery of the species in 1966, with land-clearing, development of infrastructure relating to the skiing industry and feral introduced predators exacerbating the naturally fragmented nature of their populations (Smith & Broome, 1992; Broome, 2001a; McDougall & Broome, 2007; Van der Ree et al., 2009; Schulz, Wilks & Broome, 2012b; Menkhorst et al., 2013).

The future of *Burramys parvus* is also threatened from the anticipated impact of climate change on their restricted alpine habitat (Menkhorst et al., 2013). Brereton, Bennett & Mansergh (1995) predicted a 1 °C rise in ambient temperatures will result in a loss of suitable habitat for this species, although more recent fine-scale modelling suggests that the buffering effect of the boulder field habit will preserve the bioclimatic range to some extent (Shi et al., 2015). However, because of their geographically restricted distribution, as climate change continues to impact the alpine region these possums will be unable to disperse poleward or ascend higher in elevation to avoid the increasing warmth. Additionally, increased temperatures and decreased rainfall influence food availability (Bates, 2016) and cause early snowmelt ( Hennessy et al., 2003), decreasing the survival of *B. parvus* (Broome et al., 2012).

Until recently, the distribution of *B. parvus* was thought to be restricted to the alpine and sub-alpine regions of the Bogong High Plains and Mt Buller in Victoria, and the southern region of Kosciuszko National Park in New South Wales. However, in 2010, a new population of *B. parvus* was discovered in northern Kosciuszko National Park at Happy Jacks Valley (1,200–1,310 m). This discovery was well below the once-presumed lower elevation limit for breeding populations in southern Kosciuszko National Park and Mt Higginbotham—Mt Bogong and slightly lower than the lowest populations at Mt Buller (1,300 m) ( Heinze, Broome & Mansergh, 2004; Schulz, Wilks & Broome, 2012a; Broome et al., 2013), thereby becoming the lowest elevation population currently known (Schulz, Wilks & Broome, 2012a; Bates, 2016). The new population is represented at four sites: Happy Jacks Valley (1,225 m), Snow Ridge (elevation 1,488 m), Bolton’s Hill (1,514 m) and Rough Creek (1,617 m) (Schulz, Wilks & Broome, 2012a; Bates, 2016). Some of the habitat of Happy Jacks Valley is extensively modified, and the boulder fields of the northern population are comprised of basalt, rather than the granitic substrate of the southern high elevation population (Smith & Broome, 1992; Körtner & Geiser, 1995; Broome, 2001b; Broome et al., 2013). Previous studies have demonstrated that differences in local habitat can influence the composition of the diet of *B. parvus* because they can alter the suite of arthropods and plant species available in those areas (Gibson, 2007; Gibson & Broome, 2014). Previous findings have also demonstrated a significant decrease in diversity in the diet of these possums as a function of increasing elevation, a decrease in vegetation and an increase in the abundance of Bogong Moths (*Agrotis infusa*) (Mansergh et al., 1990; Smith & Broome, 1992; Gibson, 2007; Broome et al., 2013). *A.infusa* typically comprises around 30–50% of all food consumed by *B. parvus*, being a particularly important component when individuals emerge from hibernation in spring (Broome, 2001a). Seeds and fruit of Mountain Plum Pine (*Podocarpus lawrencei)* and Snow Beard Heath (*Acrothamnus montanus)* are the main plant species present in the diet (Smith & Broome, 1992; Gibson, 2007). However, *A. infusa* has a reduced abundance at the lower elevation sites, and vegetation surveys reveal that 50% of plant species common within the high elevation sites are not present in the habitat of the new population (Smith & Broome, 1992; Gibson & Broome, 2014; Bates, 2016). Therefore, although it has been previously suggested that *B. parvus* is dependent on the regular influx of *A. infusa* and particular plant species (Broome, 2001a), the reduced availability of these food types at low elevations suggests that they may not be essential requirements.

The main purpose of this study was to determine the dietary composition of the recently discovered low elevation northern population of *B. parvus* in Kosciuszko National Park and to make comparisons between this and the higher elevation southern population. We also aimed to determine how dietary composition responded to changes in annual rainfall and how it fluctuated between seasons. Determining flexibility within the diet of *B. parvus* and investigating how changes in diet correlate with changes in rainfall and season will provide insights into the potential ability of the species to survive the environmental changes predicted to occur as climate change inexorably transforms its current habitat. On a global scale, countless species will be forced to shift their distribution due to the impacts of climate change (Thuiller, 2004). Dietary analysis of these species will enable us to assess their responses to climate change and will be critical in determining the suitability of new habitats.

**Figure 1:**
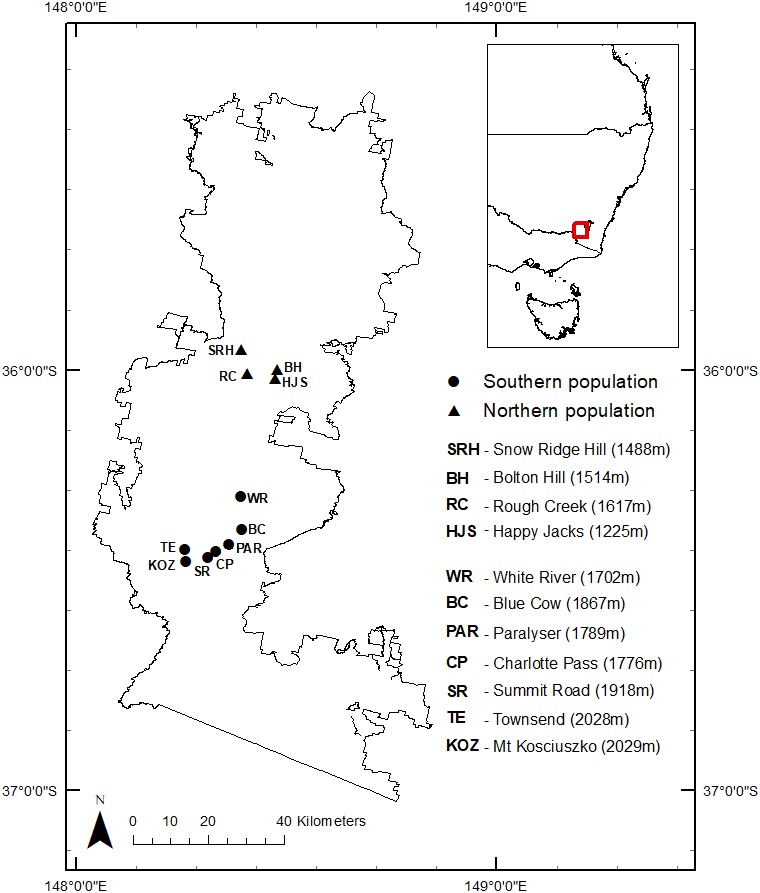
Map of Kosciuszko National Park showing sites and elevations where *B. parvus* faecal samples were collected in the southern and northern populations.

## Methods

### Study area/sample collection and design

*Burramys parvus* faecal samples from the northern population (average elevation 1,461 m) were collected during trapping surveys from 2010 to 2014. Samples from the southern population (average elevation 1,868 m) were collected between 1994 and 2014 from traps set in boulder fields as part of OEH annual monitoring surveys (project approval number 991129/01) of *B. parvus* in the Kosciuszko National Park, New South Wales, Australia (Fig. 1).

Live trapping of *B. parvus* took place using Elliot traps (33 × 10 × 10 cm, Elliot Scientific Equipment, Upwey, VIC, Australia). Traps were lined with Dacron batting and placed in a plastic bag to ensure dryness. Trapping was done over 3–4 nights at most sites. Faecal pellets were collected on the first night of trapping, using chocolate or walnut oil as an attractant to prevent contamination of faecal samples. Walnut baits were used for subsequent nights. Faecal pellets were removed from traps and placed in Eppendorf tubes for freezing. As all possums were individually marked with ear tag identification or a microchip, only one sample was taken from each individual in each sample period. Trapping effort was similar between populations with 1–4 transects and 75–100 traps per site, depending on the size of the boulder fields.

To examine dietary differences between the northern and southern populations, a total of 528 samples (from 411 individuals) were analysed: 323 samples from northern Kosciuszko sites and 205 from southern sites. All samples included in the comparative study were collected from 2011 to 2014 between the months of October and March.

To investigate differences in diet between periods of low and high rainfall, dietary results obtained from the southern sites in this study were combined with those from a dietary study of possums from the southern sites conducted in 2007 (Gibson, 2007). This resulted in a total of 791 samples (from 623 individuals) collected from 1994 to 2014 between the months of November and March. Samples from the southern population only were used to ensure no confounding effects of dietary differences between the two populations.

To determine seasonal effects on diet, 323 samples from the northern sites only were analysed to ensure that detectable differences were not confounded by regional differences in diet. Fifty-four samples were obtained between 2011 and 2014 during autumn and 269 in spring.

### Slide preparation/sample scoring

Samples were prepared using methods similar to those outlined in Gibson (2007) and Gibson, Broome & Hutchinson (2018). Two to three faecal pellets were taken from the trap samples from one individual and transferred into a 1.5 ml Eppendorf tube. Pellets were separated and crushed with forceps in 0.75 ml of 80% ethanol to ensure homogenisation. Three drops of Safranin O plant stain were added to each tube. These were shaken vigorously and left for a minimum of 8 h to facilitate staining. Samples were then washed through a 210-micron mesh sieve using an 80% ethanol solution. Material remaining at the top of the sieve was collected using forceps and transferred to a single microscope slide. Faecal material was spread to ensure a similar density between slides, facilitating comparable scoring between individual samples. Samples were left to dry before being mounted with Canada Balsam.

For each slide 10 separate random fields of view (FOV) were analysed under a light microscope at 400× magnification. Each food type was scored as present or absent within each FOV. The frequency of each food type was calculated by dividing the number of times it was scored as present by 10 (total number of FOVs analysed). This method efficiently detects the presence of major food types within faecal samples ( De Camargo et al., 2014). However, due to differential digestibility it does not quantify absolute food intake. This method assumes that observed faecal items are proportionate to the amount of that food type consumed and that food types have similar digestibility between different individuals (Gibson, Broome & Hutchinson, 2018). Plants were classified to species level. Invertebrates, because of difficulties in identifying at more precise taxonomic levels on the basis of fragmentary exoskeletons and internal organs, were classified to order, with the exception of millipedes and centipedes which could be classified to class. Morpho-species were created for food types that were common but not able to be otherwise classified.

For accurate identification, reference slides were created using known plant and invertebrate material collected from *B. parvus* trapping sites in the Kosciuszko National Park. Material was crushed with a mortar and pestle and soaked in 1M of sodium hydroxide solution for 20 min to simulate digestion. Material was stained, washed and mounted, as per methods described for faecal pellets. Photographs of all material were taken under a light microscope at 400× magnification to create a number of reference photos.

### Data analysis

Prior to analyses, food types with low abundances that could not be identified were grouped into categories by adding the frequency of all grouped food types. Invertebrate material that fit this criteria was grouped to ‘invertebrate other’ and plant material was grouped to ‘plant other’. To investigate differences in diet between periods of below average and above average rainfall, results obtained from the southern population in this study were merged with results of a previous dietary study by Gibson (2007) on the same population. Only species and orders that were identified in both studies were included in the comparative analysis. Annual rainfall was calculated by averaging rainfall data from weather stations within the southern Kosciuszko National Park study area ( Bureau of Meteorology, 2015).

To compare overall dietary composition between populations (northern, southern), rainfall regimes (above average, below average) and season (spring, autumn), we performed multivariate generalised linear models in the *mvabund* package (function manyglm, Wang et al., 2012), using binomial distribution. This method tests the matrix of all species counts as the response variable, testing whether the composition of species changes among the predictors (population, rainfall, season). Given low counts for some species, this model is the best approach as rare species contribute less to the covariance structure of the model, and therefore contribute less to the impacts of the treatment effects (Warton, Wright & Wang, 2012). We found no pattern in the distribution of residuals to indicate a poor fit of any of the ManyGLM analyses (Wang et al., 2012). We used the univariate procedure implemented in ManyGLM to examine which species/orders contributed significantly to the differences in dietary composition.

Given that low counts for rare species resulted in skewed and bimodal data, it was not appropriate to present the data as means or medians. Additionally, there were a number of individuals that had no identifiable food types in all 10 FOV’s. These individuals were included in the analyses, as a lack of identifiable food types in their diet was important in determining the overall dietary composition across each factor. For these reasons, data were displayed as the percentage of samples which had each food type.

Species richness and diversity (Shannon-Wiener index, *H*’), were calculated for each sample and compared across populations, rainfall and seasons using Wilcoxon Signed Rank tests. All statistical tests and models were performed in R (R Development Core Team, 2018).

## Results

### Dietary differences between the northern and southern populations

**Figure 2:**
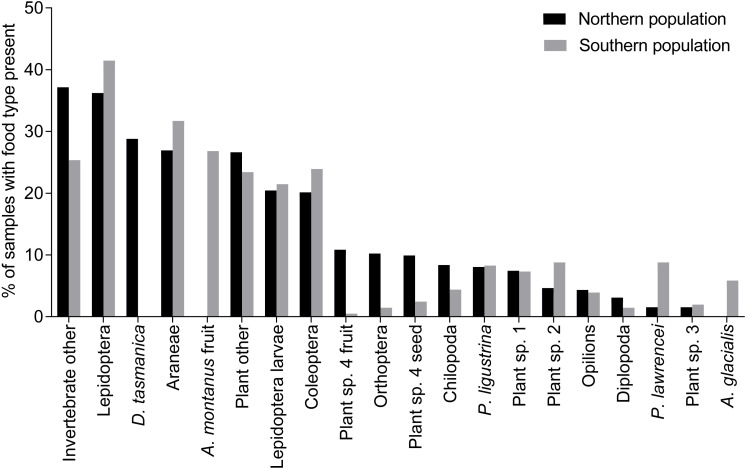
Percentage of food types in samples from northern and southern population of *B. parvus*. Percentage of *B. parvus* faecal samples that contained particuluar food types for the northern and southern population in Kosciuszko National Park.

The diet of *B. parvus* in the northern population in the Kosciuszko National Park was comprised of invertebrates, fruits and seeds, having most of the same invertebrate orders and plant species that are found in the diet of the southern population (Fig. 2). ‘Invertebrates other’ and Lepidoptera (predominantly *A. infusa*) and had the highest percentage occurrence in the samples from the northern population (Fig. 2).

Multivariate analysis suggests some evidence that the two populations had differences in overall dietary composition (*dev* = 11.83, *p* = 0.085). Invertebrates made up 64.7% of the dietary material from the northern population and 62.7% from the southern population. Lepidoptera, ‘Invertebrate other’ and Araneae were present in the highest percentage of samples for both the southern and northern population, but did not contribute significantly to differences in overall dietary composition between the two populations. The major dietary difference in plant material was the consumption of *Acrothamnus montanus* fruit and *Aciphylla glacialis* exclusively by the southern population and *Dianella tasmanica* exclusively by the northern population of *B. parvus* (Fig. 2). *Pimelea ligustrina* also contributed significantly to the differences in dietary composition (dev = 3.968, *p* = 0.046).

Average species diversity in the samples did not differ between the northern and southern populations (northern *H*′ = 0.70, southern *H*′ = 0.74, *w* = 34606.5, *p* = 0.38). This was also the case for invertebrate diversity (northern *H*′ = 0.42, southern *H*′ = 0.39, *w* = 35479.5, *p* = 0.14) and for plant diversity (northern *H*′ = 0.16, southern *H*′ = 0.20, *w* = 32201, *p* = 0.46). There was also no difference in average species richness of samples between the northern and southern populations (northern average = 2.66, southern average = 2.57, *w* = 34450.5, *p* = 0.37), nor did invertebrate species richness (northern average = 1.63, southern = 1.52, *w* = 33857.5, *p* = 0.66) and plant species richness (northern average = 0.98, southern = 0.99, *w* = 32718.5, *p* = 0.81) differ.

### Influence of rainfall on diet

**Figure 3:**
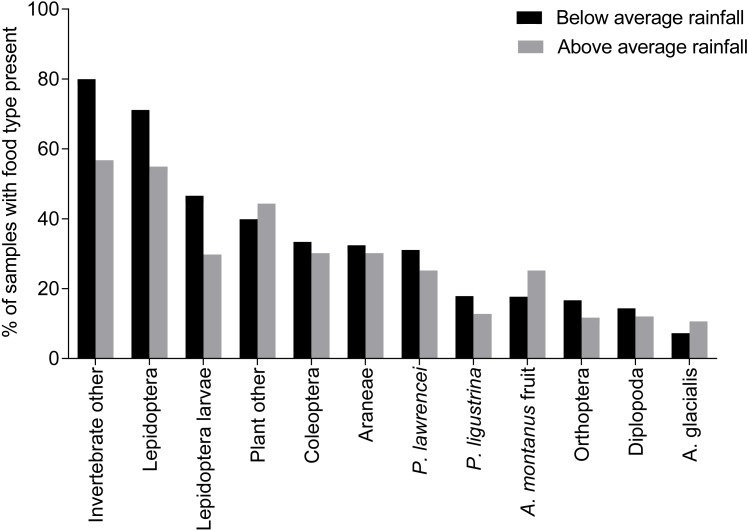
Percentage of food types in samples from below and above average rainfall years for *B. parvus*. Percentage of *B. parvus* faecal samples that contained particular food types for below average and above average rainfall years in Kosciuszko National Park.

There was a significant difference in the dietary composition during years of high rainfall and years of low rainfall (*dev* = 41.09, *p* = 0.001, Fig. 3). Invertebrates made up 79% of the dietary material for the southern population in below average rainfall years, compared with 72% in above average rainfall years.

Average species diversity was significantly higher in years of below average rainfall (below average *H*′ = 1.07, above average *H*′ = 0.88, *w* = 55972.5, *p* < 0.01). The diversity of invertebrates within the diet was also significantly higher in years of below average rainfall (below *H*′ = 0.8, above *H*′ = 0.56, *w* = 52301.5, *p* < 0.001), while plant diversity remained constant despite variations in rainfall (below *H*′ = 0.23, above *H*′ = 0.24, *w* = 72,139, *p* = 0.77).

Average species richness was significantly higher during below average rainfall years (below average = 4.15, above average = 3.31, *w* = 54,550, *p* =  < 0.001). This was also true for species richness of invertebrates (below average = 2.97, above average = 2.19, *w* = 50,750, *p* < 0.001). Plants displayed no difference in richness between years of below and above average rainfall (below average = 1.17, above average = 1.12, *w* = 68,808, *p* = 0.39).

### Effect of season on dietary composition

**Figure 4:**
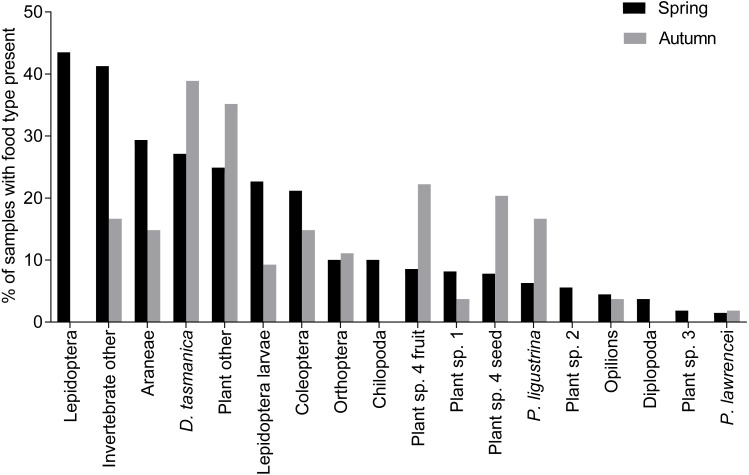
Percentage of food types in samples from spring and autumn for *B. parvus*. Percentage of *B. parvus* faecal samples that contained particular food types for spring and autumn in Kosciuszko National Park.

There was a significant difference in overall dietary composition between autumn and spring months for the northern population (dev = 31.19, *p* = 0.001, Fig. 4). In the northern Kosciuszko population, during autumn, invertebrates made up 26% of dietary material, compared to 71% during spring. Plants made up 73% during autumn and 28% in spring. Lepidopterans (*A. infusa*) were only recorded in the samples during the spring months (Fig. 4).

Species diversity was significantly higher in the spring months (autumn *H*′ = 0.5, spring *H*′ = 0.8, *w* = 5200.5, *p* < 0.001). Invertebrate diversity was also significantly higher for the spring months (autumn *H*′ = 0.15, spring *H*′ = 0.51, *w* = 4,271, *p* < 0.005), while plant diversity was higher in the autumn months (autumn *H*′ = 0.29, spring *H*′ = 0.16, *w* = 8535.5, *p* = 0.001). Average species richness (spring average = 2.71, autumn average = 1.94, *w* = 5200.5, *p* < 0.01) and invertebrate species richness (spring average = 1.86, autumn average = 0.70, *w* = 4,271, *p* < 0.001) were both significantly higher in the spring months. Plant species richness was higher during autumn (spring average = 0.84, autumn average = 1.24, *w* = 8535.5, *p* = 0.001).

## Discussion

Climate change is a global threat expected to alter the distribution and abundance of many species (McLaughlin et al., 2002). Given that the Mountain Pygmy-possum is confined to its current distribution, this assessment of diet between varying populations, rainfall amounts, and seasons, has provided critical information for determining how it might respond to future climate pressures and the suitability of new habitats.

Here we used microscopic faecal analysis to assess the diet of *B. parvus*. Methods used in this analysis were efficient for detecting the presence of major food types within faecal samples. Differential digestibility of food types is a potential source of error, as food types that are easily digested are underestimated and less digestible foods are overestimated (Putman, 1984). While this reduces the reliability of making conclusions about the total food intake of *B. parvus*, the similar digestibility of food types between individuals allows us to make accurate diet comparisons between populations, rainfall amounts and seasons.

### Dietary differences between northern and southern populations of *B. parvus*

Results indicate a similar dietary composition and no differences in species diversity and richness of diet between the northern and southern populations. The major invertebrate orders and many of the major plant species favoured by *B. parvus* were similar across both populations, and these were selectively consumed where available (Smith & Broome, 1992; Youdan, 2006; Gibson & Broome, 2014). Previous analyses of the diet of *B. parvus* in the Kosciuszko National Park have suggested a significant increase in invertebrate diversity and abundance and a decrease in plant diversity and abundance with increasing elevation (Smith & Broome, 1992; Gibson, 2007; Broome et al., 2012). Based on this conclusion, we would therefore expect differences in diversity between the northern and southern populations, given their elevation differences. However, we report no difference in species diversity between the two populations.

A plausible explanation for this could be the confounding effect of rainfall on the analysis. Samples used in the comparative study of the northern and southern populations were collected between 2011 and 2014, during a period of above average rainfall, which this study showed to be associated with a decrease in dietary diversity, thus potentially resulting in no detectable differences in the diet between the two populations during this time.

Another possible reason for failure to detect differences in species richness and diversity is that they were calculated after the data were aggregated to the categories ‘plant other’ and ‘invertebrate other’. Given that the plants and invertebrates that were grouped into categories could not be identified, it could not be determined if observable parts belonged to a single species or multiple species. As a result, this study may have underestimated the true species diversity and richness within the diet, but still this method allowed us to reliably compare our diversity and richness estimates across factors.

Multivariate analysis showed some differences between the diet of the two populations. Although there were no differences in species richness or diversity between the two populations, there was variation in the occurrence of some food types, likely a reflection of differences in local habitat and resource availability. The most notable difference between the diets was the absence of Snow Beard Heath (*Acrothamnus montanus*) in the diet of the northern population, identified in previous studies as key components of the diet of these possums and hence identified as a critical component of high elevation *B. parvus* habitats (Smith & Broome, 1992). Vegetation surveys (Bates, 2016) support this finding, suggesting *A. montanus* is absent from the sampled northern sites occupied by *B. parvus* (Bates, 2016). Plant material microscopically similar to *A. montanus* was found in the faecal remains from the northern population, but based on comparisons with reference material and habitat assessments, the plant was identified as the Tasman Flax-lily (*Dianella tasmanica),* a broad-leaved lily with violet fruits that is abundant in the habitat of the northern population, rarely occurring above the tree line (Costin et al., 2000; Bates, 2016). While *D. tasmanica* is the likely identity of the observed plant material, stable isotope analysis would improve the reliability of this identification (Kelly, 2000; Sponheimer et al., 2003; Gibson, 2007).

Previous dietary analyses have concluded that *A.infusa* moths are a critical component of the diet of *B. parvus* and an essential factor determining the abundance and survival of the species at high elevations, where at times they may constitute the only animal prey in the diet (Smith & Broome, 1992; Gibson, 2007; Schulz, Wilks & Broome, 2012a). At the lower elevation southern sites, they make up about a third of the diet in spring and 10% in autumn (Gibson, 2007; Gibson, Broome & Hutchinson, 2018). In the northern population examined in this study, *A. infusa* was absent from the diet of *B. parvus* during autumn, with 73% of the diet comprised of plants. In the absence of *A. infusa* in the habitat, the diet of this possum reflects the availability of other potential foods in the surrounding habitat, suggesting that *B. parvus* is not dependent on the regular influx of *A. infusa* provided that alternative food sources are available.

### Effect of rainfall amount on the diet of *Burramys parvus*

An increased percentage of invertebrates in the diet and an increase in invertebrate diversity and richness during low rainfall years can be attributed to an overall reduction in the availability of food, particularly seeds and fruit, in below average rainfall years (Gibson, Broome & Hutchinson, 2018). This, in turn, would result in a necessary diversification of the diet of the possums to obtain required resources (Suttle, Thomsen & Power, 2007).

It would be expected there would be a reduction in the species richness and diversity of seeds and fruit during the drier years (Knapp et al., 2002; Puig et al., 2010). Sampling methods may account for failure to detect changes in plant richness and diversity, as plant material in the faecal samples were more difficult to classify due to similarity in microscopic cell appearances. Increased difficulty in distinguishing between species likely reduced estimates of richness and diversity, concurring with previous studies which note a reduced ability to determine plant species richness and abundance from scats, due to the extensive mastication of these softer materials (Green, 1989; Jones & Barmuta, 1998).

### Effect of season on the diet of *Burramys parvus*

During autumn, *A. infusa* was absent in the diet of the northern population of *B. parvus* with 73% of the diet consisting of plants, while during spring 71% of the diet consisted of invertebrates. The changes in *A. infusa* consumption correspond to the seasonal migration of *A. infusa* and its influx into low elevations of the area during the spring months and movement to higher elevations as summer progresses (Broome, 2001a; Warrant et al., 2016). The increased consumption of invertebrates, especially *A. infusa*, may also be due to the high protein dietary requirements *B. parvus* has over the summer months, due to female reproduction and juvenile growth. (Smith & Broome, 1992; Gibson, 2007). In the absence of *A. infusa*, the diet of this possum reflects the availability of other potential foods in the habitat. This dietary flexibility suggests that these possums are less dependent on specific food resources than previously presumed.

## Conclusion

The discovery of a low altitude population of the Mountain Pygmy-possum *(B. parvus),* in combination with the demonstration of further dietary flexibility, shows that this possum may be confined to its current distribution by factors other than dietary requirements. Recent studies demonstrate that water availability is a major factor in the survival and distribution of *B. parvus* (Bates, 2016). Results presented in this study that the diet of this species is impacted by amount of rainfall supports this relatively new, water-dependent hypothesis. Considered together, the changes in rainfall associated with climate change will adversely impact the on viability of these alpine populations of *B. parvus*.

Fossil evidence suggests that ancestral species of *Burramys* occupied and in some cases were very common in lowland rainforests (Archer, 2002). Broome et al. (2012) suggest that the current distribution of *B. parvus* is probably the result of an expansion of lowland rainforests, which contained the ancestral population of *B. parvus*, into the alpine zone, during an interval of interglacial Pleistocene warming.

Fossil evidence, the discovery of the new low elevation population and this dietary analysis, collectively demonstrate that the current limited distribution of *B. parvus* is most likely to be the result of factors other than current food or habitat requirements. These other factors probably include pre-modern habitat fragmentation and water availability. Given the sensitivity of the alpine region to future climate change, it is probable that the chances for long-term survival of this possum would be extended by acclimatising and translocating a population from its current alpine habitat to potentially suitable areas of cool, temperate lowland rainforest. On the basis of results reported in the present study, it is probable that translocated individuals would opportunistically adjust to the range of previously unfamiliar foods in these lowland forests and thrive. These findings are critical for future management strategies and should provide incentive to trial translocations as a method of safeguarding the future of this critically-endangered species.

##  Supplemental Information

10.7717/peerj.6307/supp-1 Supplemental Information 1 *B. parvus* raw dietary data Raw data for dietary analysis of *B. parvus* in Kosciuszko National Park.

## Additional Information and Declarations

### Field Study Permissions

The following information was supplied relating to field study approvals (i.e., approving body and any reference numbers): Field experiments were approved by Office of Environment and Heritage project approval number 991129/01.

### Data Availability

The following information was supplied regarding data availability: Raw data are available in the Supplementary Files.
